# Both Fasting and Glucose-Stimulated Proinsulin Levels Predict Hyperglycemia and Incident Type 2 Diabetes: A Population-Based Study of 9,396 Finnish Men

**DOI:** 10.1371/journal.pone.0124028

**Published:** 2015-04-08

**Authors:** Jagadish Vangipurapu, Alena Stančáková, Teemu Kuulasmaa, Johanna Kuusisto, Markku Laakso

**Affiliations:** 1 Faculty of Health Sciences, Institute of Clinical Medicine, Internal Medicine, University of Eastern Finland, Kuopio, Finland; 2 Department of Medicine, Kuopio University Hospital, Kuopio, Finland; Joslin Diabetes Center, Harvard Medical School, UNITED STATES

## Abstract

**Background:**

Hyperproinsulinemia is an indicator of β-cell dysfunction, and fasting proinsulin levels are elevated in patients with hyperglycemia. It is not known whether proinsulin levels after a glucose load are better predictors of hyperglycemia and type 2 diabetes than fasting proinsulin.

**Methods:**

Participants were 9,396 Finnish men (mean±SD, age 57.3±7.1 years, BMI 27.0±4.0 kg/m^2^) of the population-based METabolic Syndrome In Men Study who were non-diabetic at the recruitment, and who participated in a 6-year follow-up study. Proinsulin and insulin levels were measured in the fasting state and 30 and 120 min after an oral glucose load. Area under the curve (AUC) and proinsulin to insulin ratios were calculated.

**Results:**

Fasting proinsulin, proinsulin at 30 min and proinsulin AUC during the first 30 min of an oral glucose tolerance test significantly predicted both the worsening of hyperglycemia and type 2 diabetes after adjustment for confounding factors. Further adjustment for insulin sensitivity (Matsuda index) or insulin secretion (Disposition index) weakened these associations. Insulin sensitivity had a major impact on these associations.

**Conclusion:**

Our results suggest that proinsulin in the fasting state and after an oral glucose load similarly predict the worsening of hyperglycemia and conversion to type 2 diabetes.

## Introduction

β-cell dysfunction is one of the major pathophysiological disturbances in type 2 diabetes. Proinsulin is the precursor form of insulin, synthesized in the endoplasmic reticulum, then transported to the Golgi apparatus where it is packaged into secretory vesicles, and finally cleaved to form mature insulin and C-peptide. Proinsulin accounts for 10–20% of fasting insulin in normoglycemia, but may reach values as high as 50% in patients with type 2 diabetes indicating defective processing or premature release of proinsulin by the β-cell [1–5].

High concentrations of proinsulin are observed in glucose intolerant and/or insulin resistant individuals. Elevated level of glucose is the main stimulus for increased proinsulin synthesis and secretion [6]. Prolonged exposure of β-cells to glucose results in abnormal proinsulin processing [7], which is related to the severity of hyperglycemia [3]. A β-cell defect could be either due to a primary dysfunction of the proinsulin conversion machinery (decreased activity of prohormone convertases 1/3) or a malfunction in related β-cell regulatory mechanisms that secondarily affect insulin production and secretion [4,5,8]. Recent studies have shown that common and low-frequency gene variants regulate proinsulin levels [9], and that disruption of insulin receptor expression in beta-cells leads to poor proinsulin processing by altering the expression of carboxypeptidase E enzyme [10]. The proinsulin (P) to insulin (I) ratio (P/I ratio) has been suggested to provide an additional measure of β-cell function. The fasting P/I ratio is considered as a marker of acute insulin response [11]. Disproportionate hyperproinsulinemia is recognized as an indicator of β-cell distress commonly observed in type 2 diabetes.

Fasting proinsulin levels have been associated with insulin resistance [12] and type 2 diabetes [13–16], but not with family history of diabetes [17]. However, there are no prospective studies investigating the glucose-stimulated levels of proinsulin as predictors for the worsening of hyperglycemia or conversion to type 2 diabetes. Given the fact that high proinsulin level is an indicator of beta-cell distress one would expect that glucose-stimulated proinsulin reflects even better the disturbances in glycemia than fasting proinsulin level. To investigate this question we studied the association of fasting, 30 and 120 min proinsulin levels and proinsulin area under the curve (AUC) in an oral glucose tolerance test (OGTT) with the worsening of hyperglycemia and incident type 2 diabetes in a 6-year prospective follow-up of the METSIM cohort.

## Materials and Methods

### Subjects

The study included 9,396 men from the population-based METSIM (METabolic Syndrome In Men) Study. The study protocol has been previously explained [18]. Glucose tolerance was classified according to the ADA criteria [19,20]. Among the participants, 3,033 (32.3%) had normal glucose tolerance (NGT), 4,344 (46.2%) had isolated impaired fasting glucose (IIFG), 311 (3.3%) had isolated impaired glucose tolerance (IIGT), 1,059 (11.3%) had both IFG and IGT, and 649 (6.9%) had newly diagnosed type 2 diabetes. Individuals with previously diagnosed type 1 or type 2 diabetes were excluded, and none of the participants were on anti-diabetic medication. The characteristics of the study participants are presented in Table 1.

**Table 1:** Characteristics of the METSIM study participants at baseline across the various categories of glucose tolerance.

Variable	All	NGT	IIFG	IIGT	IFG+IGT	New T2D*	P value
Number of subjects	9396	3033	4344	311	1059	649	
Age, years	57.3 ± 7.1	56.9 ± 6.9	56.8 ± 7.1	59.8 ± 7.2	59 ± 7.1	59.4 ± 6.8	**<0.001**
Body mass index, kg/m^2^	27 ± 4	25.8 ± 3.4	27 ± 3.7	27.1 ± 3.7	29 ± 4.4	29.7 ± 4.9	**<0.001**
Current smoking (%)	18.2	18	18	15.4	15.4	18.2	**0.028**
Physically active (%)	64.4	68.4	68.4	64	55.1	54.4	**<0.001**
Matsuda ISI	6.7 ± 4.2	9 ± 4.7	6.2 ± 3.3	5.8 ± 3.5	3.9 ± 2.5	3.4 ± 2.5	**<0.001**
Disposition Index	156.5 ± 74.4	211.5 ± 76.2	149.4 ± 54.1	129.6 ± 43.2	94 ± 30.7	61.4 ± 26.8	**<0.001**
OGTT fasting plasma proinsulin (pmol/l)	14.5 ± 8	11.6 ± 4.6	14.1 ± 6.4	13.9 ± 6.5	18.5 ± 9.9	24.3 ± 14.7	**<0.001**
OGTT 30 min plasma proinsulin (pmol/l)	31.4 ± 15.7	27 ± 13	32 ± 15	29.4 ± 15.1	37.3 ± 19.1	39 ± 19.2	**<0.001**
OGTT 120 min plasma proinsulin (pmol/l)	52.7 ± 28.2	42.9 ± 20.9	50.2 ± 23.9	66 ± 30.5	74.9 ± 36.3	73.4 ± 35.6	**<0.001**
OGTT fasting plasma insulin (pmol/l)	52.3 ± 39.3	37.5 ± 24.7	51.4 ± 33.4	55.3 ± 45.5	74.7 ± 48.4	89.6 ± 63.3	**<0.001**
OGTT 30 min plasma insulin (pmol/l)	401.2 ± 294.8	366.6 ± 284.2	413.1 ± 284	396.3 ± 315.8	467 ± 353.2	377 ± 275.6	**<0.001**
OGTT 120 min plasma insulin (pmol/l)	334.9 ± 345.8	222 ± 205.2	271.3 ± 246.4	578.5 ± 425.8	663.2 ± 472.5	635.2 ± 545.3	**<0.001**
Proinsulin/Insulin ratio at 0 min (%)	33.4 ± 15.6	37 ± 16.9	32.2 ± 14.3	32.2 ± 16.4	29 ± 13.5	32.3 ± 17	**<0.001**
Proinsulin/Insulin ratio at 30 min (%)	9.5 ± 4.8	9 ± 4.2	9.3 ± 4.2	9.4 ± 4.2	9.7 ± 4.4	13.4 ± 8.6	**<0.001**
Proinsulin/Insulin ratio at 120 min (%)	24.9 ± 17.3	28.1 ± 18.8	27 ± 17.5	15.3 ± 8.5	14.6 ± 8.1	17 ± 12.4	**<0.001**

Values are shown as mean ± SD for each category. P values were obtained from ANOVA for overall comparison across the five glucose tolerance categories.

* newly diagnosed individuals with type 2 diabetes at baseline

Diagnosis of type 2 diabetes at the follow-up study was based either on fasting plasma glucose (FPG) ≥7.0 mmol/L or on 2-hour plasma glucose (2hPG) ≥11.1 mmol/L in an OGTT or HbA1c ≥6.5% among 4,806 non-diabetic individuals who participated in the ongoing 5.9-year follow-up study in 2010–2014 (327 cases of new diabetes), anti-diabetic medication started between the baseline study and 31 December 2013 (N = 261 cases of new diabetes; information obtained from the National Drug Reimbursement registry), or type 2 diabetes diagnosed by physician based on medical records and/or fasting plasma glucose (FPG) ≥7.0 mmol/L, 2-hour plasma glucose (2hPG) ≥11.1 mmol/L or HbA1c ≥6.5% in outpatient/primary care laboratory measurements (N = 37 cases of new diabetes), and the lack of symptoms and signs indicating type 1 diabetes. Thus, during the follow-up a total of 625 men were diagnosed with incident type 2 diabetes. The study was approved by the Ethics Committee of the University of Eastern Finland and Kuopio University Hospital, and was conducted in accordance with the Helsinki Declaration. All study participants gave written informed consent.

### Anthropometric and other measurements

Height, weight, and hip and waist circumference were measured as previously described [18]. BMI was calculated as weight (kg) divided by height (m) squared. Smoking was classified as current smoking (yes vs. no), and physical activity as physically active (leisure time exercise at least 30 min ≥ 1 times a week vs. inactive).

### Oral glucose tolerance test

A 2-hr oral glucose tolerance test (OGTT, 75 g of glucose) was performed in the fasting state and samples for plasma glucose, insulin and proinsulin were drawn at 0, 30, and 120 min.

### Laboratory Measurements

Plasma glucose was measured by enzymatic hexokinase photometric assay (Konelab Systems reagents; Thermo Fischer Scientific, Vantaa, Finland). Insulin was determined by immunoassay (ADVIA Centaur Insulin IRI no. 02230141; Siemens Medical Solutions Diagnostics, Tarrytown, NY) having cross-reactivity with intact proinsulin of 2.6%. Proinsulin was measured by immunoassay (Human Proinsulin RIA kit Linco Research, St. Charles, MO) which measures both intact and des 31,32 split proinsulin. Cross-reactivity of proinsulin assay was 100% with intact human proinsulin, 95% with des 31,32 proinsulin, <0.1% with des 64,65 proinsulin, <0.1% with human Insulin, and <0.1% with human C-peptide. Thus our proinsulin assay includes both intact and des 31,32 split proinsulin.

### Calculations

The trapezoidal method was used to calculate the glucose, insulin and proinsulin areas under the curve (AUC) in an OGTT based on samples collected at 0, 30 and 120 min. Evaluation of insulin secretion (InsAUC_0-30_/GlucAUC_0-30_) and insulin sensitivity (Matsuda ISI) have been previously described [18,21]. Disposition index was calculated as a product of the indices of insulin sensitivity (Matsuda ISI) and insulin secretion (InsAUC_0-30_/GlucAUC_0-30_). The P/I ratios were calculated as proinsulin concentration (pmol/l) divided by the insulin concentration (pmol/l) in the fasting state (proinsulin at 0 min/Insulin at 0 min), and at 30 min (proinsulin at 30 min/Insulin at 30 min) and at 120 min (proinsulin at 120 min/Insulin at 120 min). FPG categories were generated with a 0.5 mmol/L increment in FPG levels and 2hPG categories were generated with a 1 mmol/L increase in 2hPG levels. The number of subjects per category for FPG levels was ranging from 29 to 3496 and for 2hPG levels it was 175 to 2367. FPG ≤5.0 mmol/L and 2hPG ≤5.0 mmol/L were set as the reference categories.

### Statistical analysis

Statistical analyses were conducted using IBM SPSS version 19 (IBM SPSS, Chicago, IL). All traits except for age were log-transformed to correct for their skewed distributions. All variables were standardized (based on their respective SD’s) to obtain comparable Hazard’s ratios (HR). A linear regression model was used to evaluate the proinsulin measures as predictors for Glucose AUC at the follow-up study, wherein previously diagnosed diabetes subjects were excluded from analyses. Standardized effect size (β) was estimated by linear regression analysis. To investigate the association of proinsulin measures with incident type 2 diabetes, the follow-up time was calculated (in months) and Cox regression analysis was applied. Adjustments were primarily done for age, BMI, smoking, physical activity and follow-up time where applicable. Additional adjustments were done for Matsuda ISI and Disposition index. After Bonferroni correction for multiple testing (for 14 tests given the 7 proinsulin variables and 2 glycemic traits), P<0.0036 was considered as statistically significant.

## Results

### Proinsulin levels across the categories of fasting and 2h glucose subgroups (Fig 1)

**Fig 1:**
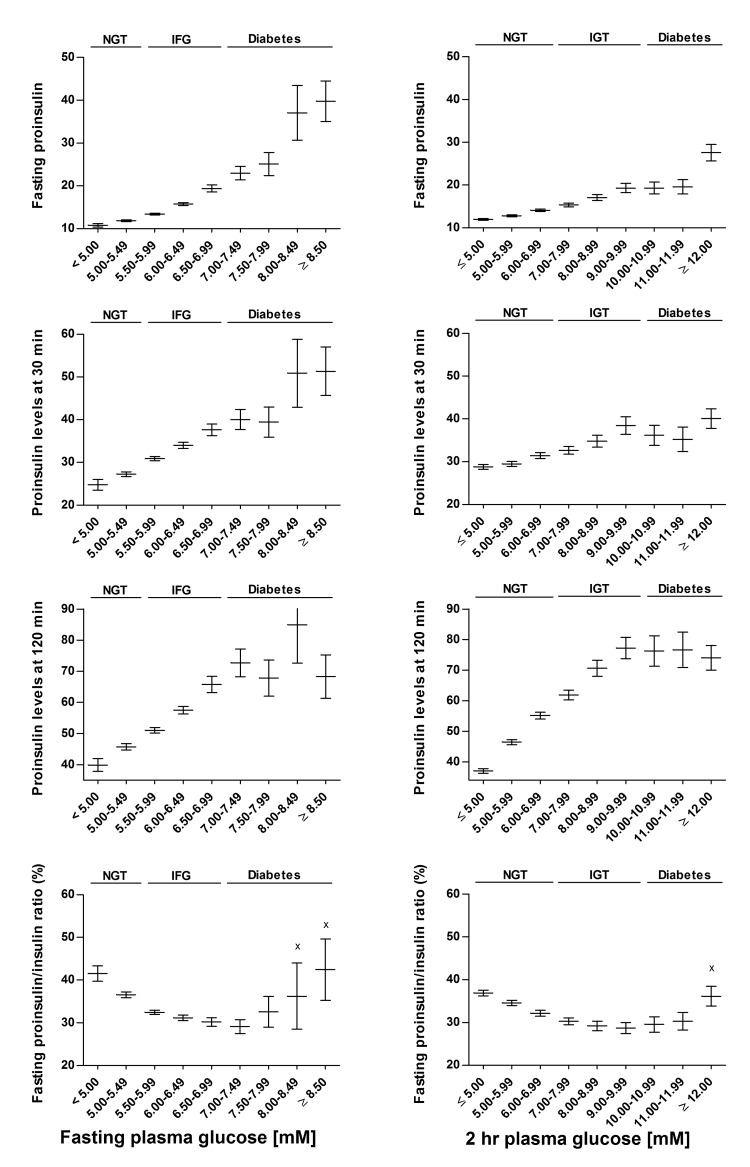
Proinsulin levels across the categories of fasting and 2 hour glucose in the METSIM cross-sectional study. Mean values of proinsulin levels (pmol/l) and their 95% confidence intervals are shown. Overall P values based on ANOVA across the categories of fasting and 2 hour glucose were significant (P<0.001) for all traits. All mean values were significantly (P<0.001) different from mean values of the reference group (FPG ≤5 mM and 2hr glucose ≤5 mM) except for those marked with ‘x’.

Across the fasting glucose subgroups (Fig 1, left panel) mean levels of fasting, 30 and 120 min proinsulin increased significantly compared with the reference category (FPG<5.00 mM). Similarly, across the 2hPG subgroups (Fig 1, right panel), fasting proinsulin levels increased significantly compared with the reference category (2hPG<5.00 mM), but 30 and 120 min proinsulin levels did not increase at 10 mM or higher glucose levels. With increasing FPG levels, the fasting P/I ratio decreased at FPG levels from 5 to 7 mM, but increased in the diabetic range (Fig 1, left panel). Similarly, across the 2hPG subgroups, the fasting P/I ratio decreased at glucose levels from 5 to 11 mM, but increased in the diabetic range (Fig 1, right panel).

### Fasting proinsulin levels across the quintiles of insulin sensitivity and insulin secretion (Fig 2)

**Fig 2:**
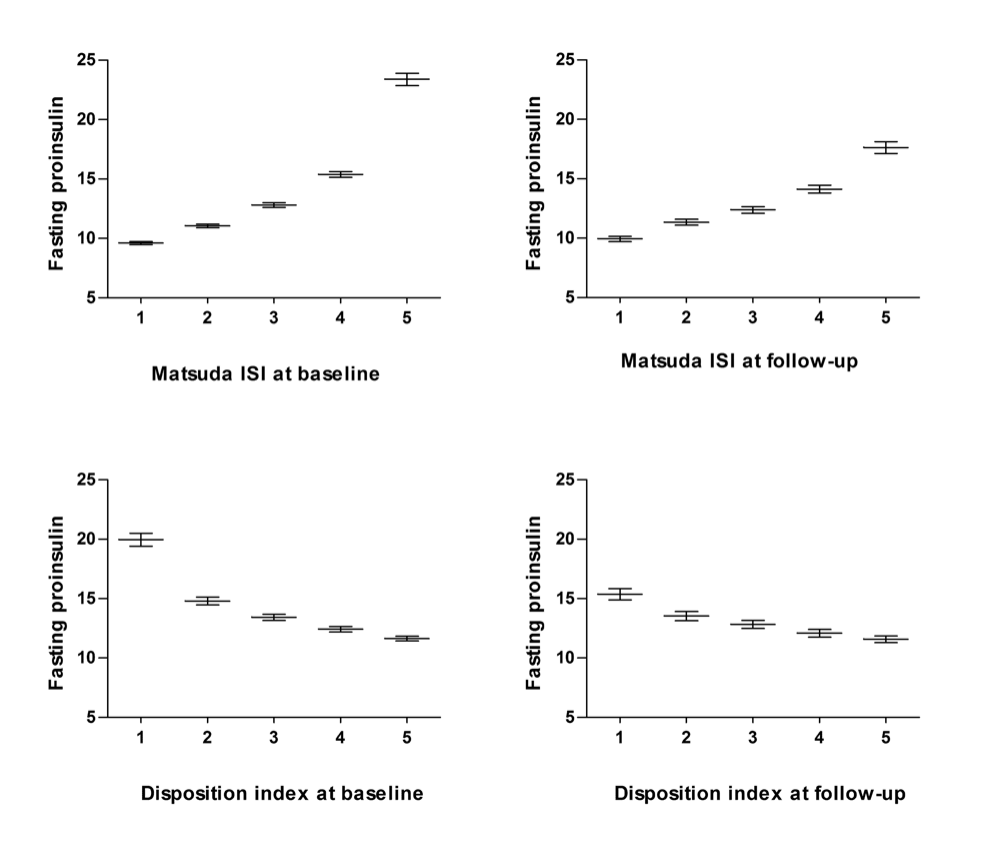
Fasting proinsulin levels across the quintiles of Matsuda ISI and Disposition index in the METSIM cross-sectional and at follow-up studies. Mean values of fasting proinsulin levels (pmol/l) at baseline and their 95% confidence intervals are shown. Overall P values based on ANOVA across the quintiles of the Matsuda ISI and the Disposition index (both baseline and follow-up) were significant, P<0.001 (Quintiles of the Matsuda ISI: 1—most sensitive 5—least sensitive; Quintiles of the Disposition index: 1—lowest value, 5—highest value).

A significant increase (P<0.0036) in fasting proinsulin levels across the quintiles of insulin sensitivity (Matsuda ISI) was observed at the cross-sectional (Fig 2, left panel) and follow-up studies (Fig 2, right panel). In contrast, fasting proinsulin levels decreased significantly across the quintiles of the Disposition index at both examinations.

### Association of proinsulin levels with the worsening of hyperglycemia (Table 2)

Fasting proinsulin levels, proinsulin levels at 30 min and 120 min in an OGTT were strongly associated with an increase in Glucose AUC at the follow-up study (P<0.0036). After adjustment for age, BMI, smoking, physical activity and follow-up time, all proinsulin measures associated significantly with Glucose AUC during the 6-year follow-up period, except for proinsulin at 30 min. The P/I ratios associated significantly with a lower Glucose AUC. After adjustment for Matsuda ISI at baseline, in addition to age, BMI, smoking, physical activity and follow-up time, the statistically significant association with Glucose AUC at follow-up was lost only for fasting proinsulin levels. Adjustment for the Disposition index resulted in a significant association of all variables with Glucose AUC except for the P/I ratio at 0 min and 120 min.

**Table 2:** Association of proinsulin levels and the proinsulin/insulin ratios measured at baseline with Glucose AUC and incident type 2 diabetes in the METSIM 5.9-year follow-up study.

Variable	Glucose AUC					Type 2 diabetes				
	β	P	P*	P*^1^	P*^2^	HR (95% CI)	P	P*	P*^1^	P*^2^
Fasting insulin	0.302	**<0.001**	**<0.001**	**<0.001**	**<0.001**	1.38 (1.33–1.43)	**<0.001**	**<0.001**	**<0.001**	**<0.001**
Fasting Proinsulin	0.233	**<0.001**	**<0.001**	0.627	**<0.001**	1.46 (1.41–1.52)	**<0.001**	**<0.001**	**<0.001**	**<0.001**
Proinsulin at 30 min	0.126	**<0.001**	0.010	**<0.001**	**<0.001**	1.36 (1.28–1.44)	**<0.001**	**<0.001**	0.026	**<0.001**
Proinsulin at 120 min	0.319	**<0.001**	**<0.001**	**<0.001**	**<0.001**	1.44 (1.38–1.50)	**<0.001**	**<0.001**	**<0.001**	**0.003**
Proinsulin AUC 0–30 min	0.163	**<0.001**	**<0.001**	**<0.001**	**<0.001**	1.44 (1.36–1.51)	**<0.001**	**<0.001**	0.769	**<0.001**
(Proinsulin / Insulin) at 0 min	-0.177	**<0.001**	**<0.001**	**<0.001**	0.801	0.79 (0.72–0.86)	**<0.001**	0.221	**<0.001**	0.005
(Proinsulin / Insulin) at 30 min	-0.001	**0.962**	**<0.001**	**<0.001**	**<0.001**	1.01 (1.03–1.09)	0.004	**<0.001**	**<0.001**	0.063
(Proinsulin / Insulin) at 120 min	-0.268	**<0.001**	**<0.001**	**<0.001**	0.097	0.48 (0.42–0.55)	**<0.001**	**<0.001**	0.005	0.661

Standardized β and P values shown were obtained from linear regression (unadjusted). P* was adjusted for age, BMI, smoking, physical activity and the follow-up time. P*^1^ was adjusted for age, BMI, smoking, physical activity, Matsuda ISI at baseline and follow-up time, P*^2^ was adjusted for age, BMI, smoking, physical activity, Disposition index at baseline and follow-up time. Unadjusted standardized Hazard’s ratio (95% CI) and P values were obtained from Cox regression. P* was adjusted for age, BMI, smoking and physical activity. P*^1^ was for adjusted for age, BMI, smoking, physical activity, and Matsuda ISI, P*^2^ was adjusted for age, BMI, smoking, physical activity and Disposition index. Number of incident cases of type 2 diabetes included in Cox regression analyses was 625. Statistically significant p-values (P<0.0036) are marked by bold.

### Association of proinsulin levels with type 2 diabetes (Table 2)

All proinsulin measures significantly predicted the development of type 2 diabetes after adjustment for age, BMI, smoking, physical activity and follow-up time, with the exception of the P/I ratio at 30 min. Fasting proinsulin level was the strongest predictor of type 2 diabetes at the follow-up study (HR = 1.46 [1.41–1.52]), both before and after adjustment for confounding factors (age, BMI, smoking, physical activity and follow-up time). It was somewhat stronger predictor of incident type 2 diabetes compared to fasting insulin. Proinsulin level at 120 min also strongly predicted the development of type 2 diabetes (HR = 1.44 [1.38–1.50]). Proinsulin AUC 0–30 min also associated with a similar HR (1.44 [1.36–1.51]). The fasting P/I ratio was associated inversely with the risk of type 2 diabetes (HR = 0.79 [0.72–0.86]), but the association was lost or became less significant after the adjustment for confounding factors. The P/I ratio at 120 min inversely associated with risk of diabetes (HR = 0.48 [0.42–0.55]).

Associations of proinsulin levels with type 2 diabetes were further examined after adjustments for additional confounding factors. After additional adjustment for Matsuda ISI fasting proinsulin, proinsulin at 120 min, the P/I ratio at 0 and 30 min remained significantly associated with type 2 diabetes (P<0.0036). When adjusted for the Disposition index, fasting proinsulin, proinsulin at 30 and 120 min and proinsulin AUC 0–30 min were significantly associated with type 2 diabetes (P<0.0036).

### Association of proinsulin levels with insulin sensitivity and insulin secretion (Table 3)

All proinsulin measures were also strongly associated with insulin sensitivity Matsuda ISI) and insulin secretion (Disposition index) at baseline and at follow-up when adjusted for age, BMI, smoking, physical activity and follow-up time, except for proinsulin at 30 min. However, additional adjustment for baseline insulin sensitivity and insulin secretion abolished all statistically significant associations at the follow-up study.

**Table 3:** Association of baseline proinsulin levels with the Matsuda ISI and the Disposition index at follow-up.

Variable	Baseline data								Follow-up data							
	Matsuda ISI				Disposition Index				Matsuda ISI				Disposition Index			
	β	P	P*	P^#1^	β	P	P*	P^#2^	β	P	P*	P^#3^	β	P	P*	P^#4^
Fasting Proinsulin	-0.660	**<0.001**	**<0.001**	**<0.001**	-0.290	**<0.001**	**<0.001**	0.007	-0.505	**<0.001**	**<0.001**	0.004	-0.230	**<0.001**	**<0.001**	0.004
Proinsulin at 30 min	-0.643	**<0.001**	**<0.001**	**<0.001**	0.030	0.005	**<0.001**	**<0.001**	-0.471	**<0.001**	**<0.001**	0.164	-0.004	0.794	**<0.001**	0.091
Proinsulin at 120 min	-0.660	**<0.001**	**<0.001**	**<0.001**	-0.392	**<0.001**	**<0.001**	**<0.001**	-0.489	**<0.001**	**<0.001**	0.044	-0.290	**<0.001**	**<0.001**	0.027
Proinsulin AUC 0–30 min	-0.680	**<0.001**	**<0.001**	**<0.001**	-0.062	**<0.001**	**<0.001**	**<0.001**	-0.506	**<0.001**	**<0.001**	0.049	-0.069	**<0.001**	0.026	0.036
(Proinsulin / Insulin) at 0 min	0.626	**<0.001**	**<0.001**	**<0.001**	0.269	**<0.001**	**<0.001**	0.016	0.451	**<0.001**	**<0.001**	0.010	0.188	**<0.001**	**<0.001**	0.158
(Proinsulin / Insulin) at 30 min	0.461	**<0.001**	**<0.001**	**<0.001**	-0.269	**<0.001**	**<0.001**	**<0.001**	0.367	**<0.001**	**<0.001**	0.131	-0.126	**<0.001**	**<0.001**	0.015
(Proinsulin / Insulin) at 120 min	0.609	**<0.001**	**<0.001**	**<0.001**	0.454	**<0.001**	**<0.001**	**<0.001**	0.452	**<0.001**	**<0.001**	0.082	0.330	**<0.001**	**<0.001**	0.006

Standardized β and P values were obtained from unadjusted linear regression. For baseline data, P* was adjusted for age, BMI, smoking and physical activity. P^#^ was adjusted age, BMI, smoking and physical activity, and additionally for 1) Disposition index or 2) Matsuda ISI. For follow-up data, P* was adjusted for age, BMI, smoking, physical activity, and follow-up time. P^#^ was adjusted for age, BMI, smoking, physical activity, follow-up time, and additionally for 3) Matsuda ISI or 4) Disposition index. Statistically significant P values (P<0.0036) are marked by bold.

## Discussion

In our study fasting, 30 min and 120 min proinsulin levels and proinsulin AUC 0–30 min significantly predicted the worsening of hyperglycemia or increased risk of incident type 2 diabetes, without any major differences between these measures. The corresponding P/I ratios were somewhat weaker predictors for hyperglycemia and the conversion to type 2 diabetes.

Impaired insulin secretion is a major pathophysiological disturbance in type 2 diabetes, and hyperglycemia further impairs β-cell function [22]. We observed that compared to the normoglycemic range, proinsulin levels significantly increased in the fasting state and at 30 min and 120 min in an OGTT with the increasing levels of FPG or 2hPG. The increase in proinsulin levels was already pronounced in the pre-diabetic state, as shown previously [23]. Mechanisms that could lead to elevated proinsulin levels indicating β-cell distress are a greater demand for insulin secretion due to hyperglycemia that depletes the mature insulin granules, the defective enzymatic proinsulin processing machinery, or the loss of β-cell viability or a combination of these mechanisms [4,5,11].

Fasting proinsulin level was a significant and consistent predictor for the worsening of hyperglycemia and the conversion to type 2 diabetes, independent of confounding factors. Previous considerably smaller studies [14–16,24], but not all [17], have reported similar findings. Proinsulin levels at 30 min and 120 min of an OGTT and proinsulin AUC 0–30 min were also significant predictors for the worsening of hyperglycemia and incident type 2 diabetes even after adjustment for insulin secretion. However, by contrast to our expectations glucose-stimulated proinsulin levels were not better predictors of incident type 2 diabetes than the fasting proinsulin level. Additionally, the adjustment for insulin sensitivity abolished the significance of the association of proinsulin at 30 min and proinsulin AUC 0–30 min with incident type 2 diabetes. This may indicate that insulin resistance, in addition to insulin secretion, plays a significant role in mediating the effects of proinsulin on the conversion to diabetes. Further evidence that insulin resistance is closely linked with proinsulin levels is our observation that proinsulin levels at baseline were more significantly associated with changes in insulin sensitivity than with insulin secretion at follow-up, although the association weakened after the adjustment for baseline measures of insulin sensitivity and insulin secretion. These results are in agreement with previous studies reporting that fasting proinsulin levels predict insulin resistance in type 2 diabetes [12].

Previous studies [23–25], but not all [26–29], have suggested that the fasting P/I ratio is increased in diabetic subjects compared to non-diabetic individuals, but we did not find significantly higher proinsulin levels in newly-diagnosed diabetic individuals. By contrast, we found a significant decrease in the fasting P/I ratio with increasing glucose levels in participants with prediabetes, as previously reported [30–32]. Conflicting results between the studies could be explained by small sample sizes in previous studies, different methods to measure proinsulin, cross-reactivity of proinsulin with proinsulin split products, and hepatic clearance of insulin [11,33,34].

In our study the fasting P/I ratio significantly predicted the worsening of hyperglycemia and incident type 2 diabetes. However, the adjustment for insulin secretion abolished this association. The P/I ratio at 120 min of an OGTT significantly predicted both hyperglycemia and type 2 diabetes after adjustment for confounding factors but lost its significance after the adjustment for the Disposition index indicating that impaired β-cell function plays a significant role in the development of hyperglycemia and conversion to diabetes in the glucose-stimulated state.

The major limitation of our study is that it includes only Finnish men. Therefore, further studies are needed whether our findings are applicable to women or other ethnic groups. There have been some suggestions that proinsulin levels relative to the C-peptide levels would be more stronger markers to reflect the degree of hyperproinsulinemia than the normal P/I ratio [31,33,35], but we did not measure C-peptide levels in our study.

In conclusion, among several proinsulin measures tested in our prospective METSIM study, fasting proinsulin, proinsulin levels at 30 min and 120 min, and proinsulin AUC 0–30 min were significant predictors for the worsening of hyperglycemia or type 2 diabetes, with fasting proinsulin being the most consistent predictor. Our study suggests that compared to fasting insulin level the measurement of glucose-stimulated proinsulin levels in an OGTT does not offer any improvement in the prediction of incident type 2 diabetes.
